# Retroviral vector-mediated hypoxia-regulated neurotrophin-3 gene transfer reduces apoptosis induced by hypoxia in PC12 cells

**DOI:** 10.1186/1750-1326-7-S1-S17

**Published:** 2012-02-07

**Authors:** Junfeng Zhang, Qindong Shi, Xinlin Chen, Pengbo Yang, Cunfang Qi, Jianshui Zhang, Haixia Lu, Lingyu Zhao, Bingqiao Zhao, Ping Zheng, Yong Liu

**Affiliations:** 1 Institute of Neurobiology, Xi’an Jiaotong University College of Medicine, #76 Yanta West Road, Xi’an, Shaanxi 710061, PR China; 2 The State Key Laboratory of Medical Neurobiology, Shanghai, 200032, PR China

## Background

Gene therapy for ischemic diseases is a prospective strategy. However, excessive expression of therapeutic genes may produce undesired side effects. Recently, multiple copies hypoxia response elements (HRE) were developed to conditionally regulate gene expression under hypoxia. As a nerve growth factor, Neurotrophin-3 (NT-3) possesses neural protect effects either in vitro or in vivo. To explore hypoxia-controlled NT-3 expression, we constructed a recombinant retrovirus vector with 5HRE and NT-3, and generated a gene transferred cell line PC12-5HRE-NT3 to determine effects of conditionally expressed NT-3 on apoptosis induced by hypoxia in PC12 cells.

## Method

Five copies of HRE from human VEGF gene and simian virus 40 minimal promoter (SV40mp) were employed to construct a cassette and neurotrophin-3 was inserted into its downstream to generate RV-5HRE-NT3. Mediated by retrovirus, 5HRE-NT3 was transferred into PC12 cells and screened with G418, gene transferred cell lines were generated and identified by reporter gene EGFP, Reverse transcription PCR (RT-PCR) and immunofluorescence cell staining. Conditional expression of NT-3 was detected by RT-PCR, ELISA and Western blot. Apoptosis induced by hypoxia was evaluated by TUNEL. Apoptosis related molecules, phosphorylated p38 and active Caspase-3 were assayed by Western blot.

## Results

We successfully constructed the retroviral vector RV-5HRE-NT3 and generated the gene transferred cell line PC12-5HRE-NT3. Under normoxia, NT-3 expressed at an extremely low level in PC12-5HRE-NT3, whereas it significantly increased under hypoxia (P < 0.05). There was no significant difference in PC12-NT3 between normoxia and hypoxia. Conditionally expressed NT-3 reduced apoptosis induced by hypoxia in gene transferred cell line PC12-5HRE-NT3(P < 0.05) but not PC12-5HRE-EGFP or PC12 cells. Additionally, activation of p38 and Caspase-3 were depressed in PC12-5HRE-NT3 under hypoxia, which indicated that both of them were involved in the protective effect against apoptosis.

## Conclusions

These results suggest that 5HRE-SV40mp is an ideal cassette to regulate therapeutic gene expression in response to hypoxia, and 5HRE-NT3 can be further developed as an effective therapeutic gene to provide neuroprotection against cerebral ischemia.

